# *Listeria monocytogenes* growth kinetics in refrigerated ready-to-eat dips and dip components

**DOI:** 10.1371/journal.pone.0235472

**Published:** 2020-06-30

**Authors:** Joelle K. Salazar, Vidya Natarajan, Diana Stewart, Megan Fay, Lauren J. Gonsalves, Tanvi Mhetras, Chinmyee Sule, Mary Lou Tortorello

**Affiliations:** 1 Division of Food Processing Science and Technology, U. S. Food and Drug Administration, Bedford Park, Illinois, United States of America; 2 Institute for Food Safety and Health, Illinois Institute of Technology, Bedford Park, Illinois, United States of America; University of Bologna, ITALY

## Abstract

Refrigerated ready-to-eat (RTE) dips often have pH and water activity combinations conducive to the proliferation of foodborne pathogens, including *Listeria monocytogenes*. This study conducted product assessments of five refrigerated RTE dips: baba ghanoush, guacamole, hummus, pesto, and tahini, along with individual dip components including avocado, basil, chickpeas, cilantro, eggplant, garlic, and jalapeno pepper. Dips and dip components were inoculated with 2 log CFU/g of *L*. *monocytogenes* and stored at 10°C for 28 days. The pathogen was enumerated throughout storage and growth rates were determined using the DMFit program to compute the time required for *L*. *monocytogenes* to achieve a 1 log CFU/g increase in population. Survival and growth rates varied significantly between the refrigerated RTE dips and dip components assessed in this study. For dips, *L*. *monocytogenes* progressively decreased in baba ghanoush, pesto, and tahini. In contrast, the pathogen proliferated in both hummus and guacamole and the highest growth rate was observed in guacamole (0.34±0.05 log CFU/g per day) resulting in a 1 log CFU/g increase in population in 7.8 days. *L*. *monocytogenes* proliferated in all dip components with the exception of eggplant and garlic. The pathogen achieved the highest growth rate in chickpeas (2.22±1.75 log CFU/g per day) resulting in a computed 1 log CFU/g increase in only 0.5 days. Results from this study can aid in understanding how *L*. *monocytogenes* behaves in refrigerated RTE dips and dip components and data can be utilized in understanding product formulations and in risk assessments.

## Introduction

Foodborne recalls and outbreaks in the U.S. are becoming more prevalent for ready-to-eat (RTE) refrigerated dips, sauces, and spreads including hummus and guacamole. The formulations of these foods generally have a water activity (a_w_; >0.92) and pH (>4.4) which are within growth ranges for foodborne microorganisms. Due to the intrinsic properties, individual product assessments would be required to determine to what extent these foods support the growth of foodborne pathogens under refrigeration control. Since *Listeria monocytogenes* can proliferate in food matrices during refrigerated storage, product assessments of refrigerated RTE dips with this pathogen are essential.

From 2002 to 2017, there have been 17 human foodborne illness outbreaks associated with hummus in the U.S., resulting in a combined 1105 illnesses, 64 hospitalizations, and 4 deaths [1]. All four of the deaths reported were due to two outbreaks of listeriosis in 2013. One of the outbreaks of foodborne illness due to *Listeria monocytogenes*-contamination of hummus occurred in California and sickened 28 individuals which led to 25 hospitalizations and 3 deaths. The other listeriosis outbreak due to hummus was multistate and sickened 8 people and led to 7 hospitalizations and one death.

In addition to foodborne outbreaks, there have been 22 recalls from 2010 to 2019 due to hummus or hummus-containing food products [2]. Out of these recalls, 17 (89%) were due to possible contamination by *L*. *monocytogenes*. In 2016, one company initiated a voluntary recall of various hummus products in the U.S. and Canada after routine environmental testing revealed that 18 swabs near food handling equipment were positive for *L*. *monocytogenes* [3]. One of the isolated strains of *L*. *monocytogenes* matched a strain found in a retail hummus product from 2015, indicating that the particular strain of the pathogen may be persistent in the processing environment. More recently, in 2019, another company recalled 91 different types of hummus over concerns of contamination with *L*. *monocytogenes* [4]. Some of the varieties of hummus recalled included roasted garlic, red pepper, black bean, cilantro jalapeno, basil pesto, and sesame pine nut.

Recalls associated with other types of refrigerated RTE dips have also occurred. Two types of guacamole, original and spicy, were recalled in 2017 due to possible contamination with *L*. *monocytogenes* [5]. The pathogen was detected in two packages during routine testing. Baba ghanoush was also recalled in 2017 due to *L*. *monocytogenes* contamination of the production environment [6]. In the same year, pesto made with basil was recalled due to potential *L*. *monocytogenes* contamination [7]. Additionally, although generally not refrigerated, tahini (cooked and blended sesame seeds) which is an ingredient in hummus and baba ghanoush, has been the subject of 10 recalls in the span of only a year between 2012 and 2013 [2]; although not associated with *L*. *monocytogenes*, these recalls suggest the potential for contamination with this pathogen. Commonly, outbreaks of listeriosis and recalls of RTE foods are typically due to contamination of the food product from the environment during manufacture and processing or during packaging [8–10], but can also occur due to individual ingredient contamination [11, 12]. If *L*. *monocytogenes*-contaminated ingredients are used in the manufacture of hummus or other refrigerated RTE dips, the final product may also be contaminated if no kill step is utilized on the final product.

While studies have assessed the prevalence and survival of *L*. *monocytogenes* in RTE products including meats [13–16], seafood [15–18], dairy products [19–21], and vegetables [22–24], less information is known about how this pathogen persists in RTE refrigerated dips. For example, two studies have determined that *L*. *monocytogenes* is capable of growth in hummus when stored at refrigeration (4°C) [25, 26]. However, another study concluded that *L*. *monocytogenes* does not grow in hummus stored at 4°C [27]; the discrepancy in the latter results may be due to the formulation of the commercially-available hummus which was obtained from a food manufacturing company and used in the study. More data are needed to determine under what conditions *L*. *monocytogenes* is capable of survival and growth in these types of food matrices. The refrigerated RTE dips of interest included hummus, guacamole, tahini, baba ghanoush, and pesto. This research assessed the growth and survival of *L*. *monocytogenes* in these refrigerated RTE dips as well as in the individual components of these dips.

## Materials and methods

### *L. monocytogenes* strains and inoculum preparation

The *L*. *monocytogenes* strains utilized were 806 (isolated from hummus), 3132 (isolated from avocado), 0352 (isolated from cream cheese), and ScottA (clinical isolate [28]) and were rifampicin resistant [29]. After culturing at 37°C for 16–18 h in Brain Heart Infusion broth (BHI; Thermofisher Scientific, Waltham, MA) with 200 μg/mL rifampicin, individual cultures were normalized to an OD_600_ of 0.8 and washed twice with Butterfield’s Phosphate Buffer (BPB; Thermofisher Scientific, Waltham, MA). The cultures were combined to form a four-strain cocktail of 9 log CFU/mL, which was verified by serial dilution and plating onto Brain Heart Infusion agar (BHIA; Thermofisher Scientific, Waltham, MA) with 200 μg/mL rifampicin.

### Preparation of dip components and dips

All ingredients were sourced from local retail grocers and online distributors. Ingredients from multiple brands were combined for each trial. Dips (baba ghanoush, guacamole, hummus, pesto, and tahini) were prepared as described previously [29] and the formulations of each dip are listed in Table 1. Dip components were also prepared as previously described [29]. Briefly, boiled chickpeas were combined with sterile water and blended (Magic Bullet, NutriBullet LLC, Pacoima, CA) for 1 min to produce mashed chickpeas. Mashed eggplant was prepared by roasting the eggplants at 232°C in a conventional oven for 45 min, removing the skins, and blending for 1 min. Mashed avocado was prepared by removing the avocado skins and mashing the flesh by hand for 5 min. Basil (leaves only), cilantro, garlic (skins removed), and jalapeño pepper (seeds removed) were chopped by hand using a sterile knife and cutting board.

**Table 1:** Ingredients in the ready-to-eat (RTE) dips prepared in this study.

Dip	Ingredients
Baba ghanoush	mashed, roasted eggplant (77% w/w), tahini (10% w/w), olive oil (7% w/w), lemon juice (5% w/w), salt (1% w/w)
Hummus	mashed chickpeas (70% w/w), tahini (10% w/w), water (7% w/w), olive oil (7% w/w), lemon juice (5% w/w), salt (1% w/w)
Guacamole	mashed avocado (71% w/w), diced tomato (10% w/w), diced onion (8% w/w), lemon juice (5% w/w), diced jalapeño pepper (3.5% w/w), chopped cilantro (1.5% w/w), salt (1% w/w)
Pesto	olive oil (49% w/w), grated parmesan cheese (24% w/w), ground pine nuts (20% w/w), ground basil leaves (5% w/w), minced garlic (1% w/w)
Tahini	ground toasted sesame seeds (70% w/w), olive oil (30% w/w)

### Water activity and pH measurements

The water activity (a_w_) of each freshly-prepared dip and dip component was measured using a water activity meter (4TE, Aqualab, Decagon, WA) and 1-g samples. For pH, 5-g samples were homogenized with 5 mL of sterile water and the pH of the homogenate was measured with a pH meter (220, Mettler Toledo, OH). Both a_w_ and pH were measured in triplicate samples for each of 3 independent trials.

### Inoculation and storage of dip components and dips

Mashed chickpeas, eggplant, and avocado (2000 g each) were inoculated with the *L*. *monocytogenes* cocktail at approximately 2 log CFU/g and mixed by hand for 5 min. Fifty-g portions of chopped basil, cilantro, garlic, and jalapeño were spot inoculated (10 spots of 10 μL each) resulting in approximately 2 log CFU/g and dried at ambient for 1 h. After baba ghanoush, guacamole, hummus, pesto, and tahini were prepared according to formulation (Table 1), they were each inoculated with the *L*. *monocytogenes* cocktail at 2 log CFU/g. After inoculation, the dips and dip components were stored in 50-g portions in 8 oz deli-style containers with lids at 10°C abuse temperature for up to 28 d. Additional inoculated mashed avocado, chickpeas, and tahini (2000 g each) were stored at 10°C for 7 d for use in the preparation of dips with contaminated ingredients.

### Preparation of dips containing contaminated ingredients

Hummus and guacamole dips were also prepared using selected contaminated ingredients (avocado, chickpeas, and tahini) that had been inoculated and then stored at 10°C for 7 d. Contaminated mashed avocado was used to prepare guacamole dip. Contaminated mashed chickpeas or contaminated tahini was used to prepare hummus. Dips were stored in 50-g portions in 8 oz deli-style containers with lids at 10°C for up to 28 d.

### Enumeration and enrichment of *L*. *monocytogenes*

After 0, 1, 3, 7, 10, 14, 21, and 28 d, triplicate 50-g samples of each dip and dip component were transferred to stomacher bags and homogenized with 50 mL Buffered *Listeria* Enrichment broth (BLEB; Becton, Dickinson and Company, Sparks, MD), serially diluted, and plated onto Plate Count agar (PCA; Becton, Dickinson and Company, Sparks, MD) with 200 μg/mL of rifampicin. When plate counts were below the level of enumeration (1.70 log CFU/g), samples were also enriched using the FDA BAM procedure [30].

### Growth kinetics computation and statistical analysis

Growth kinetics (growth rates and lag phases) were computed based on the Baranyi and Roberts model [31] using DMFit version 3.0 (Institute of Food Research, Norwich, UK) add-on for Excel available from ComBase (www.combase.cc). The time to a 1 log CFU/g increase was calculated by using the inverse of the growth rate, taking into account lag phases, where applicable. Differences in initial *L*. *monocytogenes* populations, growth rates, and ending populations were statistically analyzed using ANOVA and p-value <0.05 was considered significant. Due to the low starting inoculation (~2 log CFU/g), the initial populations of *L*. *monocytogenes* on all RTE dips and dip components included estimates (<25 CFU) and these estimates were also included to compute growth kinetics.

## Results

### pH and a_w_ values of refrigerated RTE dips and dip components

The pH and water activities for all refrigerated RTE dips and dip components are depicted in Table 2. For the RTE dips, pH values ranged from 4.27±0.92 (baba ghanoush) to 5.83±0.28 (tahini). The pH of tahini was significantly higher than any other RTE dip. For dip components, pH values ranged from 5.06±0.06 (eggplant) to 6.85±0.27 (cilantro). The pH of cilantro and avocado (6.82±0.12) were significantly higher than any other dip component or RTE dip. Values for a_w_ were not significantly different for all RTE dips and dip components with the exception of pesto (0.891±0.007) and tahini (0.164±0.030) which were both significantly lower. Besides pesto and tahini, a_w_ values ranged from 0.977±0.029 (hummus prepared with contaminated chickpeas) to 0.998±0.001 (jalapeño pepper).

**Table 2:** pH and a_w_ values for the ready-to-eat (RTE) dips and dip components prepared in this study.

RTE dip or dip component	pH±SD	a_w_±SD
Baba ghanoush	4.27±0.92 ^a^	0.980±0.022 ^a^
Guacamole	4.82±0.12 ^ab^	0.991±0.005 ^a^
Hummus	5.12±0.14 ^b^	0.987±0.003 ^a^
Pesto	4.87±0.40 ^b^	0.891±0.007 ^b^
Tahini	5.83±0.28 ^c^	0.164±0.030 ^c^
Avocado	6.82±0.12 ^d^	0.996±0.003 ^a^
Basil	6.00±0.59 ^c^	0.982±0.005 ^a^
Chickpeas	6.00±0.50 ^c^	0.989±0.002 ^a^
Cilantro	6.85±0.27 ^d^	0.995±0.004 ^a^
Eggplant	5.06±0.06 ^b^	0.996±0.006 ^a^
Garlic	6.03±0.07 ^c^	0.985±0.002 ^a^
Jalapeño pepper	6.18±0.15 ^c^	0.998±0.001 ^a^
Guacamole: *contaminated avocado*^1^	5.00±0.03 ^b^	0.981±0.001 ^a^
Hummus: *contaminated chickpeas*^1^	4.65±0.02 ^ba^	0.977±0.029 ^a^
Hummus: *contaminated tahini*^1^	5.00±0.02 ^b^	0.989±0.017 ^a^

SD, standard deviation

Different lowercase letters indicate significant difference between pH or a_w_ (columns) between all dips and dip components

^1^ Component inoculated at 2 log CFU/g *L*. *monocytogenes* and then stored at 10°C for 7 d prior to dip preparation

### Population dynamics of *L*. *monocytogenes* in refrigerated RTE dips

**Fig 1:**
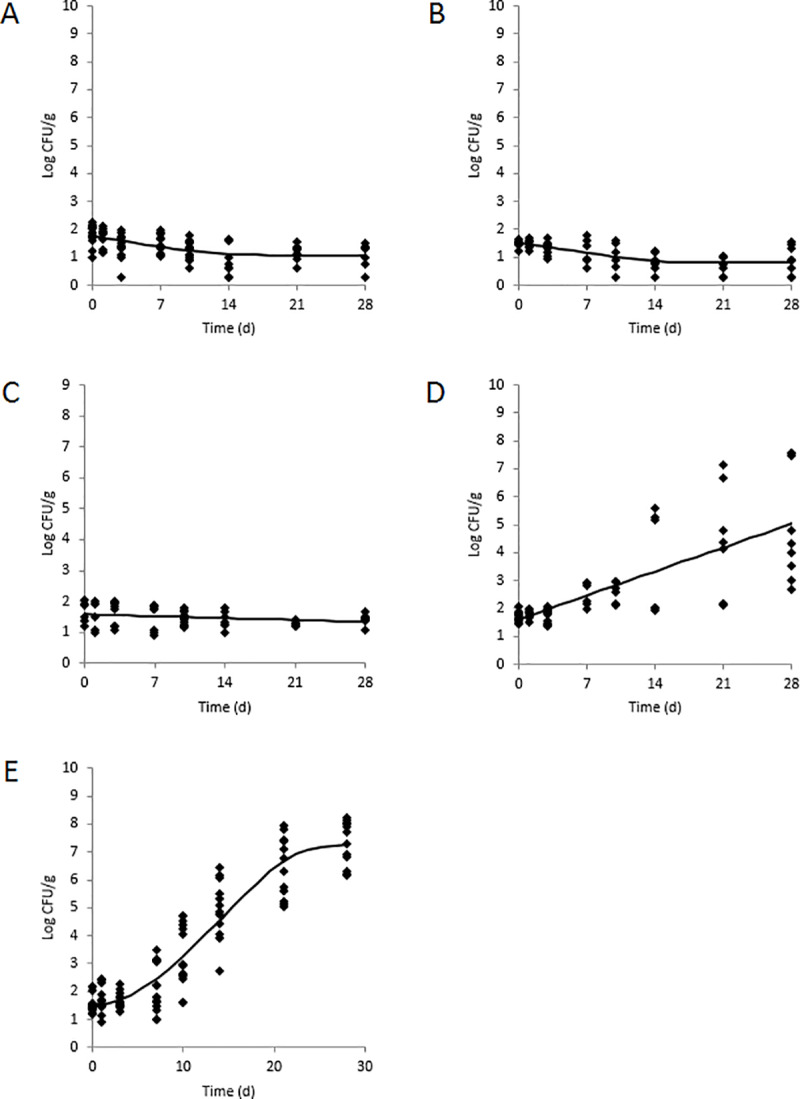
Population dynamics of *L*. *monocytogenes* in A) baba ghanoush, B) tahini, C) pesto, D) hummus, and E) guacamole during 28 d storage at 10°C. Observed data (black circles) with standard deviations (n = 9) and predicted growth models (solid black lines) are shown.

The initial inoculated population levels of *L*. *monocytogenes* in the five refrigerated RTE dips were not significantly different and ranged from 1.49±0.12 to 1.81±0.37 log CFU/g (Fig 1 and Table 3). After 28 d storage at 10°C, populations of *L*. *monocytogenes* decreased to <1.70 log CFU/g in baba ghanoush (pH 4.27, a_w_ 0.980), pesto (pH 4.87, a_w_ 0.891), and tahini (pH 5.83, a_w_ 0.164), but the pathogen was still detected in enrichments of all samples. The calculated growth rates of the pathogen in these three RTE dips were negative and would correspond to a 1 log CFU/g reduction in *L*. *monocytogenes* after 20.0 days for baba ghanoush and tahini and after 100.0 days for pesto. On the other hand, *L*. *monocytogenes* proliferated in both guacamole and hummus during 28 d storage with growth rates of 0.34±0.05 and 0.16±0.01 log CFU/g per d, respectively. Based on the growth rate, the pathogen would achieve a 1 log CFU/g increase in population in hummus in 6.3 d. For guacamole, based on the growth rate and taking into consideration the computed lag phase (4.9 d), a 1 log CFU/g increase would occur in 7.8 d. No other lag phases were computed. *L*. *monocytogenes* obtained a significantly higher population after 28 d in guacamole compared to hummus (7.31±0.80 vs 5.95±1.76 log CFU/g, respectively).

**Table 3:** Growth kinetics of *L*. *monocytogenes* in ready-to-eat (RTE) dips and dip components during 28 d storage at 10°C.

RTE dip or dip component	Initial population (log CFU/g±SD)	Growth rate ((log CFU/g)/d±SE)	Lag phase (d±SE)	Ending population (log CFU/g±SD)	Time (d) to a 1 log CFU/g increase	*r*^2^
Baba ghanoush	1.81±0.37 ^ae^	-0.05±0.01 ^a^	ND	<1.70 (+)	NA	0.29
Guacamole	1.58±0.31 ^a^	0.34±0.05 ^b^	4.88±1.59	7.31±0.80 ^ad^	7.8	0.87
Hummus	1.71±0.18 ^ae^	0.16±0.01 ^c^	ND	5.95±1.76 ^b^	6.3	0.73
Pesto	1.66±0.35 ^a^	-0.01±0.01 ^d^	ND	<1.70 (+)	NA	0.06
Tahini	1.49±0.12 ^af^	-0.05±0.01 ^a^	ND	<1.70 (+)	NA	0.43
Avocado	1.29±0.16 ^adf^	0.67±0.03 ^f^	ND	8.25±0.59 ^a^	1.5	0.97
Basil	2.43±0.93 ^e^	0.18±0.01 ^c^	ND	6.93±0.50 ^d^	5.6	0.82
Chickpeas	1.88±0.28 ^a^	2.22±1.75 ^g^	1.09±1.60	7.70±0.74 ^ad^	0.5	0.89
Cilantro	2.49±0.61 ^e^	0.20±0.08 ^bc^	ND	3.55±2.07 ^c^	5.0	0.27
Eggplant	1.85±0.29 ^a^	-0.03±0.02 ^ad^	ND	2.41±1.00 ^c^	NA	0.05
Garlic	1.37±0.72 ^f^	-0.09±0.03 ^h^	ND	<1.70 (+)	NA	0.27
Jalapeño pepper	2.11±0.64 ^ae^	0.28±0.02 ^b^	ND	6.91±0.24 ^d^	3.6	0.93
Guacamole: *contaminated avocado*^1^	6.36±0.29 ^b^	-0.17±0.02 ^e^	ND	<1.70 (+)	NA	0.79
Hummus: *contaminated chickpeas*^1^	4.53±0.71 ^c^	0.09±0.02 ^c^	ND	6.49±0.33 ^b^	11.1	0.34
Hummus: *contaminated tahini*^1^	0.63±0.43 ^d^	0.26±0.09 ^bc^	ND	2.27±0.23 ^c^	3.8	0.72

SD, standard deviation

SE, standard error

NA, not applicable

ND, no lag phase was determined

*r*^2^, coefficient of determination

(+), *L*. *monocytogenes* was detected in all enrichments

Different lowercase letters indicate significant difference between initial populations, growth rates, or ending populations (columns) between all dips and dip components

^1^ Component inoculated at 2 log CFU/g *L*. *monocytogenes* and then stored at 10°C for 7 d prior to dip preparation

### Population dynamics of *L*. *monocytogenes* in dip components

**Fig 2:**
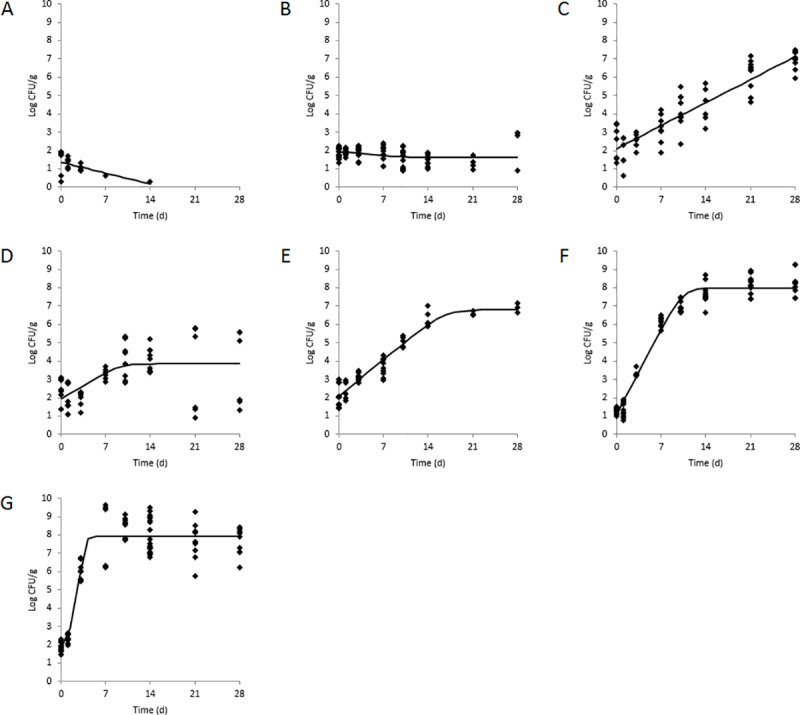
Population dynamics of *L*. *monocytogenes* in dip components including A) chopped garlic, B) mashed eggplant, C) chopped basil, D) chopped cilantro, E) chopped jalapeño pepper, F) mashed avocado, and G) mashed chickpeas during 28 d storage at 10°C. Observed data (black circles) with standard deviations (n = 9) and predicted growth models (solid black lines) are shown.

Generally, the initial populations of *L*. *monocytogenes* in the dip components were similar and ranged from 1.29±0.16 (avocado) to 2.49±0.61 (cilantro) (Fig 2 and Table 3). *L*. *monocytogenes* proliferated on all dip components during 28 d storage at 10°C with the exception of eggplant and garlic. For garlic, the *L*. *monocytogenes* population was <1.70 log CFU/g after 14 d storage, whereas the population of the pathogen on eggplant after 28 d was 2.41±1.00 log CFU/g. Growth rates of *L*. *monocytogenes* in garlic and eggplant were negative and would correspond to a 1 log CFU/g reduction in population after 11.1 and 33.3 d, respectively. *L*. *monocytogenes* proliferated on all other dip components during 28 d storage at 10°C with growth rates ranging from 0.18±0.01 log CFU/g per d (basil) to 2.22±1.75 log CFU/g per d (chickpeas). *L*. *monocytogenes* achieved significantly higher growth rates on avocado (0.67±0.03 log CFU/g per d) and chickpeas compared to all other dip components, corresponding to 1 log CFU/g increases in population after 1.5 d for avocado and only 0.5 d for chickpeas (including the computed lag phase of 1.1 d). *L*. *monocytogenes* populations after 28 d storage were highest on avocado, basil, chickpeas, and jalapeño pepper (8.25±0.59, 6.93 ± 0.50, 7.70±0.74, and 6.91±0.24 log CFU/g, respectively). It should be noted that DMFit computed a positive growth rate with a low *r*^2^ value for cilantro (0.20) due to the high standard deviation of the *L*. *monocytogenes* population data at 21 and 28 d.

### Survival of *L*. *monocytogenes* in refrigerated RTE dips prepared with contaminated ingredients

**Fig 3:**
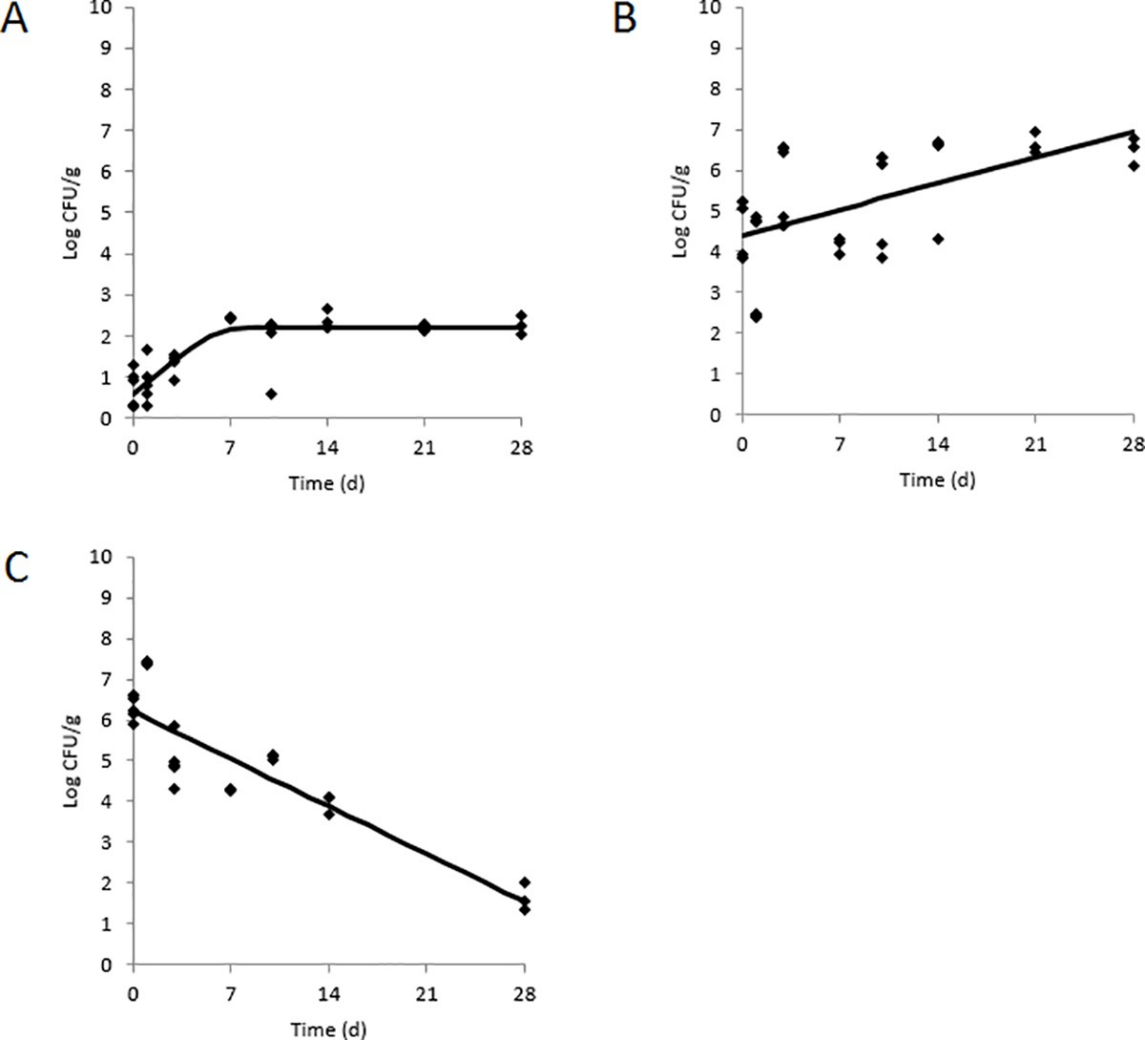
Survival of *L*. *monocytogenes* in hummus prepared with A) contaminated tahini or B) contaminated mashed chickpeas, and in C) guacamole prepared with contaminated mashed avocado during 28 d storage at 10°C. Observed data (black circles) with standard deviations (n = 9) and predicted growth models (solid black lines) are shown.

After 7 d storage at 10°C, the populations of *L*. *monocytogenes* in avocado, chickpeas, and tahini were 6.11±0.27, 7.89±1.74 and 1.20±0.46 log CFU/g, respectively (Fig 2). After this 7 d storage, the contaminated ingredients were used to prepare either guacamole or hummus. The previously contaminated and stored avocado was used to prepare guacamole and the previously contaminated and stored chickpeas or tahini was used to prepare hummus. During 28 d storage at 10°C, *L*. *monocytogenes* survived but did not proliferate in guacamole prepared with the contaminated avocado (Table 3 and Fig 3). The resulting growth rate was negative and the population after 28 d was below the level of enumeration (<1.70 log CFU/g), although still detectable after enrichment. In contrast to the guacamole which was prepared without contaminated ingredients and supported a 5.7 log CFU/g increase of *L*. *monocytogenes* after 28 d, the pathogen decreased 4.7 log CFU/g in guacamole made with the contaminated avocado even though the pH and a_w_ were similar; this difference was most likely due to the high initial level of *L*. *monocytogenes* in the avocado (6.11 log CFU/g) which was used to prepare the guacamole. For the hummus dips, the growth rates of *L*. *monocytogenes* were 0.09±0.02 and 0.26±0.09 log CFU/g per d for hummus prepared with contaminated chickpeas or tahini, respectively. No lag phases for *L*. *monocytogenes* were computed in these two dips. Based on the growth rates, *L*. *monocytogenes* would therefore achieve a 1 log CFU/g population increase in 11.1 or 3.8 d. Compared to hummus, *L*. *monocytogenes* had a significantly higher growth rate in the hummus prepared with contaminated tahini. However, the maximum population of 2.27±0.23 log CFU/g was achieved after only 7 d and the population was not significantly different from 7 to 28 d. It should be noted that DMFit computed a positive growth rate with a low *r*^2^ value for the hummus prepared with contaminated chickpeas (0.34) due to the high *L*. *monocytogenes* population at 3 d compared to 1 and 7 d.

## Discussion

This study examined the survival of *L*. *monocytogenes* in refrigerated RTE dips including baba ghanoush, guacamole, hummus, pesto, and tahini, as well as associated dip components. All products assessed in this study with the exception of pesto, tahini, and baba ghanoush, had pH and a_w_ combinations that theoretically would support the growth of foodborne pathogens. Therefore, product assessments were conducted for all refrigerated RTE dips and dip components with emphasis on growth kinetic data including growth rates and resulting times for a 1 log CFU/g increase in *L*. *monocytogenes*.

Overall, this study determined that *L*. *monocytogenes* did not proliferate in baba ghanoush, pesto, or tahini at 10°C abuse temperature. The average pH and a_w_ values (4.27 and 0.980, respectively) indicated that baba ghanoush would possibly not be an environment conducive to the growth of *L*. *monocytogenes* but may allow the pathogen to survive for extended periods of time. Using the formulation for baba ghanoush in this study, it was determined that *L*. *monocytogenes* survived during 28-day storage at 10°C but a 1 log CFU/g reduction in population was computed after 20 days. Other formulations of baba ghanoush with different concentrations of either lemon juice or citric acid have been examined for their capabilities of supporting *Salmonella enterica*, *E*. *coli* O157:H7, and *Staphylococcus aureus* [32, 33]. Results have determined that after baba ghanoush (pH 3.9–5.8) was contaminated with either *S*. *enterica* or *E*. *coli* O157:H7, both pathogens rapidly declined but were still present for >7 days at 4, 10, and 21°C [33]. Eggplant, the main component of baba ghanoush, had an average pH and a_w_ combination (5.06 and 0.996, respectively) favorable to the proliferation of *L*. *monocytogenes* in this study. However, the pathogen survived in eggplant stored at 10°C and a 1 log CFU/g reduction in population in 33.3 days was computed. The lack of proliferation of *L*. *monocytogenes* in eggplant may be due to competition with resident microbiota in the food matrix or due to other intrinsic factors. In addition, *L*. *monocytogenes* also survived but did not proliferate in tahini, another component of baba ghanoush.

Tahini, cooked and blended sesame seeds, is RTE and is stored at ambient or refrigeration temperatures. Tahini is comprised largely of lipids (57–65%) and proteins (23–27%) [34] and has a very low a_w_ (0.164). Overall, it is not surprising that *L*. *monocytogenes* did not grow in this RTE dip, however a 1 log CFU/g population reduction was computed after only 20 days. Similar studies have assessed the fate of foodborne pathogen survival in tahini including *E*. *coli* O157:H7, *S*. *enterica*, and *L*. *innocua* (a surrogate for *L*. *monocytogenes*) [34–36]. Results determined that *E*. *coli* O157:H7 and *L*. *innocua* survived in tahini (pH 5.6, a_w_ 0.31) during 28-day storage at 10, 21, and 37°C, although populations of *L*. *innocua* decreased approximately 1–3 log CFU/g [34]. Although tahini may not be a favorable environment for *L*. *monocytogenes* for proliferation, using contaminated tahini as an ingredient in other refrigerated RTE dips with higher water activities (with the exception of baba ghanoush as determined in this study) may lead to the proliferation of this pathogen.

The survival of *L*. *monocytogenes* in hummus, a refrigerated RTE dip comprised mainly of chickpeas and tahini, was also determined in this study. *L*. *monocytogenes* proliferated in hummus during 28-day storage at 10°C and was computed to increase by 1 log CFU/g in hummus after 6.3 days. Similar results have been reported in the literature [25, 37]. *L*. *monocytogenes* was determined to increase approximately 2 log CFU/g in hummus after 10-day storage at 4 or 10°C; the hummus formulation (pH 6.45) consisted of boiled chickpeas, tahini, and water [37]. *L*. *innocua* was also determined to increase by approximately 3 log CFU/g in a hummus formulation (pH 6.28, a_w_ 0.983) of boiled chickpeas, tahini, water, and salt during 15-day storage at 4°C [25]. However, a study examining the survival of *L*. *monocytogenes* in commercially-prepared hummus (pH 4.53) containing preservatives (sodium bisulfite, sodium benzoate, and potassium sorbate) determined that the pathogen did not grow during 27-day storage at 4 or 10°C [27]. The pH along with the preservatives present in the hummus formulation most likely contributed to the lack of growth of the pathogen.

Hummus was also prepared in this study with either contaminated chickpeas or tahini to determine if the source of contamination played a role in *L*. *monocytogenes* survival. Chickpeas are the main component in hummus and are boiled in water and mashed before use. In chickpeas, *L*. *monocytogenes* achieved the highest growth rate (2.22 log CFU/g per d) of any refrigerated RTE dip or dip component in this study, resulting in a 1 log CFU/g increase in only 0.5 days during 10°C storage. When hummus was prepared with either contaminated chickpeas or tahini, *L*. *monocytogenes* reached a 1 log CFU/g increase in population during storage after 11.1 and 3.8 days, respectively. As the contaminated chickpeas and tahini used to prepare the hummus were already stored for 7 days at 10°C, the initial levels of *L*. *monocytogenes* in the two hummus dips were significantly different (4.53 vs 0.63 log CFU/g, respectively), which may have impacted the resulting growth rates. With a lower initial population of *L*. *monocytogenes* in the hummus prepared with contaminated chickpeas, a higher growth rate and shorter time to a 1 log CFU/g increase may be expected.

Pesto is comprised generally of basil, pine nuts, parmesan cheese, olive oil, and garlic, although other herbs or nuts can be used. *L*. *monocytogenes* did not proliferate in the refrigerated RTE pesto formulation prepared in this study (pH 4.87, a_w_ 0.891), but did survive, which is consistent with other studies [38, 39]. Similar results were observed with *S*. *enterica* in pesto (pH 5.5, a_w_ 0.28–0.30) during four-day storage at 4°C where the rate of decline of the pathogen was 0.29 log CFU/g per day [40]. *L*. *monocytogenes* survival was also examined in two components of pesto, basil and garlic, in this study. Results determined that the pathogen survived but did not grow on garlic during 28-day storage at 10°C, which was expected as garlic has antimicrobial properties [26, 41]. Basil, however, was capable to supporting the growth of *L*. *monocytogenes*; the pathogen increased 1 log CFU/g on basil after 5.6 days. Limited information exists on the survival of *L*. *monocytogenes* on herbs. One study examined how *L*. *monocytogenes* survived on intact basil plants and determined that the pathogen survived during 28-day storage at ambient [42]. Similarly, both *S*. *enterica* and *E*. *coli* O157:H7 have been reported to survive on basil for 19 days at 4°C [43].

The formulation of the refrigerated RTE guacamole used in this study included avocado, tomato, onion, jalapeño pepper, cilantro, lemon juice, and salt, although many other ingredients can be used. In this study, *L*. *monocytogenes* proliferated in the guacamole (pH 4.82, a_w_ 0.991) during 28-day storage at 10°C and achieved the highest growth rate (0.34 log CFU/g per d) out of all refrigerated RTE dips examined. Studies on the survival of *L*. *monocytogenes* in guacamole are sparse. One study determined that the *L*. *monocytogenes* population in commercially produced processed guacamole containing preservatives (pH 5.3, a_w_ 0.98–0.99) did not change during 15-day storage at 4 or 7°C [44]. Avocado, cilantro, and jalapeño pepper, components of guacamole, were also analyzed in this study. *L*. *monocytogenes* was capable of growth in all three components during 28-day storage at 10°C and was computed to achieve 1 log CFU/g population increases in avocado, cilantro, and jalapeño pepper in 1.5, 5.0, and 3.6 days. The proliferation of *L*. *monocytogenes* in avocado pulp has previously been described [23, 44]. *L*. *monocytogenes* proliferated in commercially prepared avocado pulp (pH 6.7, a_w_ 0.98–0.99) stored at 4 or 22°C; at 22°C, the pathogen increased 4 log CFU/g after only 3 hours [44]. Similarly, a 1 log CFU/g increase in *L*. *monocytogenes* population was observed in cut avocado pulp (pH 6.23, a_w_ 0.992) after 5–8 hours at 25°C [23]. *L*. *monocytogenes* survival has also been previously assessed in both cilantro and jalapeño pepper [42, 45]. On whole, damaged, and internally inoculated jalapeño pepper, *L*. *monocytogenes* survived during 14-day storage at 7 or 12°C [45]; the pathogen increased approximately 2 log CFU/g in internally inoculated jalapeño pepper after 14 days at 7°C. In this study, *L*. *monocytogenes* increased by 1 log CFU/g on jalapeno after 3.6 days, however the temperature for storage was higher (10°C). On cilantro plants, *L*. *monocytogenes* did not grow but survived for 28 days at ambient [42]. Interestingly, *L*. *monocytogenes* had similar growth rates on the two herbs assessed in this study (cilantro and basil). However, the pathogen reached a plateaued average population on 3.55 log CFU/g on cilantro after 14 d storage, whereas the population increased on basil to an average of 8.25 log CFU/g after 28 d. it is possible that the native microbiota on the cilantro may have hindered *L*. *monocytogenes* proliferation beyond 14 d.

Overall, *L*. *monocytogenes* survived in all of the refrigerated RTE dips and dip components examined in this study. Both guacamole and hummus dips at the formulations tested supported the growth of *L*. *monocytogenes* at 10°C. Avocado, basil, chickpeas, cilantro, and jalapeño pepper also supported the growth of this pathogen. Since available data on the survival of *L*. *monocytogenes* in refrigerated RTE dips are sparse, the growth kinetic data generated in this study may be useful for understanding the risk of *L*. *monocytogenes* in these products and can aid in risk assessments. The data support reformulation to adjust pH and/or the addition of preservatives in addition to cold chain management during storage of the dip components and the RTE dips.
